# L-Glutamine therapy reduces endothelial adhesion of sickle red blood cells to human umbilical vein endothelial cells

**DOI:** 10.1186/1471-2326-5-4

**Published:** 2005-07-25

**Authors:** Yutaka Niihara, Neil M Matsui, Yamin M Shen, Dean A Akiyama, Cage S Johnson, M Alenor Sunga, John Magpayo, Stephen H Embury, Vijay K Kalra, Seong Ho Cho, Kouichi R Tanaka

**Affiliations:** 1 Department of Medicine, Harbor-UCLA Medical Center, UCLA School of Medicine, Torrance, CA USA; 2 Department of Pediatrics, Harbor-UCLA Medical Center, UCLA School of Medicine, Torrance, CA USA; 3 Department of Medicine, San Francisco General Hospital, UCSF School of Medicine, San Francisco, CA USA; 4 Department of Biochemistry, USC Keck School of Medicine, Los Angeles CA USA; 5 Medicine/Hematology, USC Keck School of Medicine, Los Angeles CA USA

## Abstract

**Background:**

We have previously demonstrated that therapy with orally administered L-glutamine improves nicotinamide adenosine dinucleotide (NAD) redox potential of sickle red blood cells (RBC). On further analysis of L-glutamine therapy for sickle cell anemia patients, the effect of L-glutamine on adhesion of sickle RBC to human umbilical vein endothelial cells (HUVEC) was examined.

**Methods:**

The first part of the experiment was conducted with the blood samples of the 5 adult sickle cell anemia patients who had been on L-glutamine therapy for at least 4 weeks on a dosage of 30 grams per day compared to those of patient control group. In the second part of the experiment 6 patients with sickle cell anemia were studied longitudinally. Five of these patients were treated with oral L-glutamine 30 grams daily and one was observed without treatment as the control. t-test and paired t-test were used for determination of statistical significance in cross-sectional and longitudinal studies respectively.

**Results:**

In the first study, the mean adhesion to endothelial cells with the autologous plasma incubated cells were 0.97 ± 0.45 for the treated group and 1.91 ± 0.53 for the nontreated group (p < 0.02). Similarly with lipopolysaccharide (LPS) incubated cells the mean adhesion to endothelial cells were 1.39 ± 0.33 for the treated group and 2.80 ± 0.47 for the untreated group (p < 0.001). With the longitudinal experiment, mean decrease in the adhesion to endothelial cells was 1.13 ± 0.21 (p < 0.001) for the 5 treated patients whereas the control patient had slight increase in the adhesion to endothelial cells.

**Conclusion:**

In these studies, oral L-glutamine administration consistently resulted in improvement of sickle RBC adhesion to HUVEC. These data suggest positive physiological effects of L-glutamine in sickle cell disease.

## Background

Sickle cell disease is a devastating hereditary disorder with excruciating morbidities often resulting in the premature demise of the patients. This disease affects primarily those of African descent, although other ethnic groups such as the Hispanic population on American continents are also affected with relatively high incidence [1-3].

Incapacitating complications of sickle cell disease are usually due to severe anemia and frequent vasoocclusive processes which damage tissues [1-6]. The cause of these events is attributed largely to increased adherence of sickle red blood cells to vascular endothelium[4,7,8]. With alteration in adherence to endothelial cells, sickle RBC will have a longer transit time through capillaries which leads to vasoocclusion with accumulation of less deformable deoxygenated sickle RBC [7-9]. Thus, amelioration of pathologic RBC adherence is considered to be an important target in therapy of sickle cell disease.

In the current article, the effect of L-glutamine therapy on sickle RBC adherence to HUVEC cells is presented. L-glutamine is a precursor to NAD[10,11]. Previous studies have shown that oral L-glutamine therapy improves NAD redox potential of sickle RBC[10]. Also, among those patients treated with L-glutamine, there were subjective reports of improvement in clinical paramenters such as chronic pain and energy level[10].

The study presented here was conducted in an attempt to better understand mechanism by which L-glutamine may potentially exert a beneficial effect on sickle RBC. In the cohort that we have studied, oral L-glutamine therapy consistently led to reduced adhesion of sickle RBC to HUVEC in static assays with both cross-sectional and longitudinal studies.

## Methods

### Cross-sectional study

#### Reagents

LPS (lipopolysaccharide) preparation (*Escherichia coli *0111:B4), Hanks' buffered saline solution, bovine serum albumin (BSA), HEPES buffer and all other chemicals were from Sigma Chemicals Co (St Louis, MO). Six-wells tissue culture plates and Falcon Primaria tissue culture flasks were from Fisher scientific (Pittsburgh, PA). Sodium-^51^Chromate (^51^Cr) and Aquasol scintillation fluid were obtained from NEN (Dupont NEN, Wilmington, DE).

#### Cell cultures

HUVEC were harvested from umbilical cord veins by collagenase digestion, as previously described[12]. Endothelial cells were identified by their cobblestone morphology, immunofluorescence staining with factor VIII-related antigen and uptake of diacetylated low-density lipoprotein (Biomedical Technologies, Stoughton, MA). Cells were passaged by trypsinization with 1% trypsin-EDTA (GIBCO BRL, Grand Island, NY) every 4–5 days. HUVEC were used from passages 2 to 6.

#### Patients, control and blood samples

Blood samples were drawn from homozygous sickle cell patients living in the Los Angeles area or from volunteer control subjects after obtaining informed consent as approved by the Harbor-UCLA Committee on Human Research. All subjects were in steady state condition without acute conditions including painful crisis. Nine adult sickle cell anemia patients 18 years and older participated. The treatment group consisted of 5 patients (mean age of 38.8 ± 7.8 with range of 30 to 47) who had been on L-glutamine therapy continuously for 8 weeks or longer at the dosage of 30 grams orally per day. The sickle cell control group consisted of 4 patients (mean age of 29.3 ± 9.0 with range of 20 to 37) who had not been on L-glutamine or any other anti-sickling therapy for at least a year. One of the patients in the non-treatment group participated at two separate time points as sickle cell control. Normal control samples were drawn from healthy volunteers.

#### ^51^Cr labeling of RBC

Five to 7 ml of heparinized blood from normal donors and sickle cell subjects were centrifuged at 450 × g for 20 min and plasma was saved in a refrigerator and the buffy coat (leukocytes and platelets) was discarded. The pellet was washed three times in Hanks' buffered saline solution with centrifugation at 450 × g for 5 minutes and then RBC were suspended to 20% hematocrit in 5 ml HBSS (Hanks' buffered saline solution). RBC in HBSS were incubated with 10 μCi/ml ^51^Cr for 1 hr at 37°C with 5% CO_2_.

#### Static assay for the adherence of RBC to cultured HUVEC

^51^Cr-sodium chromate-labeled RBC were washed three times with 10 vol of HBSS and used at a hematocrit of 2% in HBSS. HUVEC grown to confluence in 6-well plates were washed twice with HBSS (3 ml) followed by the addition of ^51^Cr-labeled RBC (hct 2%) and HBSS to a final volume of 1 ml. Five percent autologous platelet-free plasma (3 wells) or 100 ng/ml of LPS (3 wells) was added and the contents were incubated for 45 minutes at 37°C with 5% CO_2_. Nonadherent RBC were removed by aspiration and monolayers were washed three times with 1 mL of HBSS containing 0.5% BSA.200 ul lysis buffer was added to each well and lysis solution was transferred to scintillation vial. 5 μl RBC was added to 200 μl lysis buffer and the solution was used as the baseline value. Two μl of scintillation solution were added to each vial and the radioactivity in the cell monolayer-containing adherent RBC was determined and the percentage of RBC adherent was calculated[8,13].

### Statistical analysis

The ratio of percent adhesion of patient samples to that of normal control was obtained for each sample. The statistical significance of difference of the ratio between the treatment groups was determined using t-test.

### Longitudinal study

#### Reagents

Bovine serum albumin, Hanks' buffered saline solution, calcium/magnesium-free phosphate-buffered saline, electron microscopic grade glutaraldehyde, and HEPES buffer were from Sigma Chemicals Co (St Louis, MO). Human umbilical vein endothelial cells, subculture reagent package (Trypsin EDTA, HEPES Buffered Saline Solution, Trypsin Neutralizing Solution) and endothelial cell growth medium (EGM) including all supplements and growth factors were from Clonetics Corp (San Diego, CA). Two-chambered culture slides, Falcon Primaria tissue culture flasks were from Fisher Scientific (Pittsburgh, PA).

#### Cell culture

Cells were grown at 37°C in 5% CO_2_/95% air in EGM at 85% confluence; the cells were subcultured to 2 chambered slides. The second to the fourth passages were used for adherence assays no later than 24 hours after HUVEC had reached confluence.

#### Patients, control and blood samples

Blood samples were drawn from homozygous sickle cell patients of the Harbor-UCLA Medical Center or from volunteer control subjects after obtaining informed consent as approved by the Harbor-UCLA Committee on Human Research. All subjects were in steady state condition, none had received blood transfusions within 4 months, and none had history of treatment with hydroxyurea. Six adult sickle cell anemia patients 18 years of age and older participated. Five patients (mean age 40.0 ± 16.4 with range of 19 to 57) were treated for 4 to 8 weeks with oral L-glutamine at dosage of 30 grams a day. One patient (49 years old) who served as sickle cell control had no treatment. The whole blood samples were drawn at baseline which was within 4 weeks of initiation of therapy and then after 4 to 8 weeks of therapy with L-glutamine for the 5 patients. The blood samples of untreated sickle cell control patient were drawn along with the treatment patients at baseline and then 8 weeks later. For each assay, normal control blood was also drawn from normal volunteers.

#### Gravity adherence assays

This method of assaying static adherence of RBC to HUVEC monolayers was adapted from a published method[14]. Heparinized blood from normal donors and sickle cell subjects was centrifuged at 5000 × g for 5 minutes and plasma was saved in the refrigerator and buffy coat (leukocyte and platelets) was discarded. RBC were washed three times in Hanks' buffered saline solution, centrifuged at 1000 × g for 5 minutes. RBC were then centrifuged at 5000 × g and suspended to 3.5% hematocrit in HAH buffer (Hanks' buffered saline solution, 1% BSA, 50 mmol/L HEPES pH 7.4) containing 17% autologous platelet-free plasma from the heparinized blood sample. Confluent HUVEC monolayers were washed with HAH buffer to remove traces of plasma. After washing with HAH buffer, each well was filled with 0.8 ml HAH and one well was treated with LPS (200 ng/ml) and incubated at 37 C, 5%CO2, 95% humidity for 1 hr. After treating cells with LPS for 1 hr, the monolayers were covered with sickle cell (SS) RBC suspensions and incubated at 37°C for 25 minutes. The wells were then filled completely with HAH, sealed with packing tape, and inverted at 37°C for 20 minutes. While still inverted, the well walls and gaskets of the slide chambers were removed. The slides were rinsed twice with HAH buffer to remove nonadherent RBC, fixed in 3% glutaraldehyde, stained, and mounted. RBC adherence was monitored visually by microscopy at 100× magnification and quantified by counting RBC adherent to HUVEC monolayers in 16 fields marked by a grid (same random fields for each sample). The mean adherence and standard error of mean (SEM) for each sample were calculated and results were used to determine the reported means from at least four experiments, only when the SEM of each sample was less than 20% of the means. Results were analyzed by the Student's t-test, with which p < 0.05 was considered significant.

### Statistical analysis

The ratio of mean endothelial adhesion RBC of patient samples to that of normal control was obtained for each sample. The statistical significance of difference of the ratio between the baseline and after treatment was determined using paired t-test.

## Results

### Cross-sectional study

**Figure 1:**
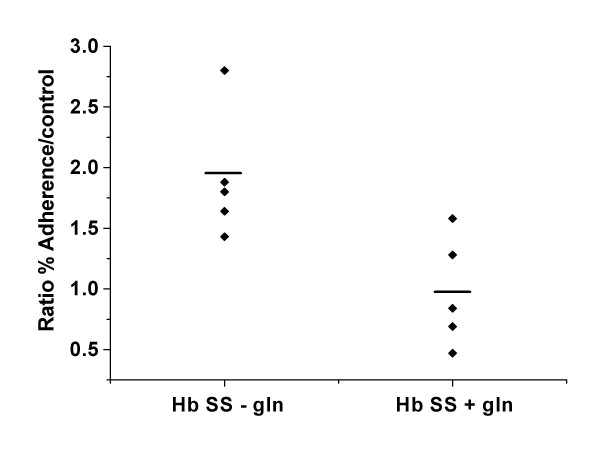
Ratios of HUVEC adhesion rates of sickle RBC to those of normal RBC for the treatment group (0.97 ± 0.45) and non-treatment group (1.91 ± 0.53) when the cells were incubated with autologous plasma alone. (p < 0.02)

**Figure 2:**
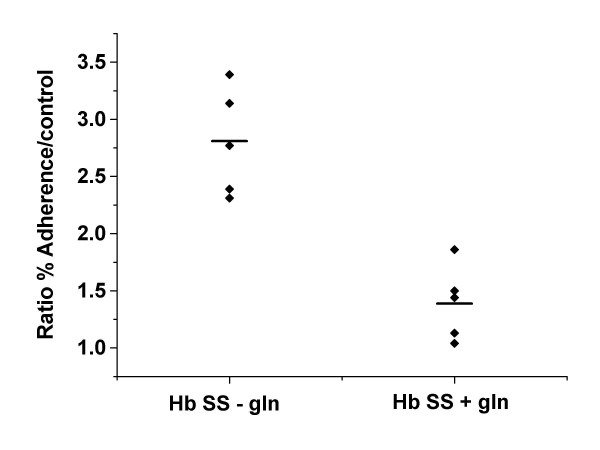
Ratios of HUVEC adhesion rates of sickle RBC to those of normal RBC for the treatment group (1.39 ± 0.33) and non-treatment group (2.80 ± 0.47) when the cells were incubated with autologous plasma and LPS. (p < 0.001)

The mean ratios with assays using autologous plasma and LPS incubated cells were 0.97 ± 0.45 and 1.39 ± 0.33, respectively for the L-glutamine treated group (N = 5). For the untreated group the mean ratios were 1.91 ± 0.53 and 2.80 ± 0.47 for autologous plasma and LPS incubated cells respectively (N = 4 with one of the patients studied twice at two time points) The decreased adhesion to endothelial cells by the L-glutamine treated group were statistically significant in both assays with autologous plasma incubated cells (p < 0.02) and LPS incubated cells (p < 0.001) (Figures 1 and 2). The endothelial adhesion of RBC from non-treated sickle cell patients were as expected and several fold higher than that of normal control. On the other hand, the results of the treatment group were consistently similar to that of the normal control, thus resulting in ratio of close to 1 for both assays using LPS or autologous plasma only. Because of the relatively small size of cohorts, there was a trend for age difference in the ages of the two groups, mean age of 38.8 ± 7.8 with range of 30 to 47 for the treatment group and 29.3 ± 9.0 with range of 20 to 37 for the non-treatment group. However, there was no statistical significance with p > 0.1.

### Longitudinal study

**Figure 3:**
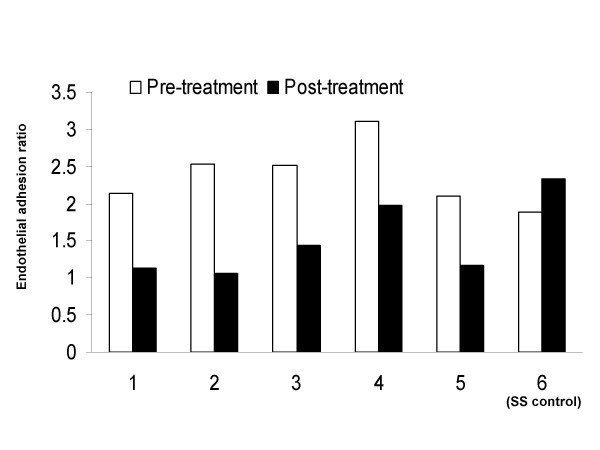
Ratios of HUVEC adhesion rates of sickle RBC to those of normal RBC before and after treatment for the five sickle cell anemia patients with one sickle cell anemia control patient who was not treated. Mean decrease of the adhesion ratios was 1.13 ± 0.21 with p < 0.001 for the five treated patients, whereas the control patient actually had slight increase in the adhesion ratio over the same period.

In contrast to the cross-sectional study, for this portion of experiment, each patient became his/her own reference and provided baseline value. The results were consistent with the data of cross-sectional study noted above. Compared to the baseline value, the endothelial adhesion of sickle RBC decreased in each patient (N = 5) within 4 to 8 weeks of oral L-glutamine therapy. The mean decrease was 1.13 ± 0.21 with p < 0.001. For the patient who participated as sickle cell control, the sickle RBC adhesion to endothelial cells increased slightly compared to the baseline (Figure 3).

## Discussion

The vaso-occlusive process is mediated primarily by congestion and closure of the microvasculature with rigid sickle RBC[4,8,15]. It is well described that the oxygenated sickle RBC is relatively deformable but it starts to become less so upon unloading of oxygen molecules to tissue[16]. The duration of time required for sickle RBC to become less deformable is thought to be more than the usual capillary transit time for RBC[7,9]. Therefore, had it not been for the prolonged capillary transit time, the sickle RBC would have already exited the microvascular system before it becomes less deformable and trapped in the microvasculature. Thus, factors that affect the capillary transit time become crucial in initiation and propagation of vaso-occlusive event.

When Hebbel and colleagues first reported an increase in endothelial adhesion of sickle RBC two decades ago, one of the major factors involved in the pathophysiology of vasoocclusion started to unfold[8]. Since then, several endothelial adhesion molecules which are abnormally expressed in both RBC and endothelial cells of sickle cell disease patients have been described[7,14,17,18]. Also, involvement of leukocytes, platelets and inflammation in general with endothelial adhesion has been described[4,19,20].

In the study presented in this article, endothelial adhesion of sickle RBC was used as the primary end point to evaluate the efficacy of L-glutamine therapy. Although clinical outcome measurements, such as the incidence of sickle cell crises or mortality rate cannot be replaced for endothelial adhesion of sickle RBC, it may be a reasonable surrogate in predicting clinical efficacy of an experimental therapeutic modality.

A role for L-glutamine in sickle cell disease was conceived when Zerez and colleagues looked closely into the redox process in sickle RBC focusing on NAD which is a potent antioxidant[21]. L-glutamine is a precursor for NAD. In sickle RBC, NAD metabolism is altered favoring an increase in the synthesis of NAD but with decreased NAD redox potential[21] As NAD is a major antioxidant molecule in RBC, the phenomenon of increased synthesis for NAD with decreased NAD redox potential was interpreted as a compensatory mechanism of sickle RBC in the presence of increased oxidant stress[21]. The presence of increased oxidant stress and oxidant susceptibility of sickle cell has been described independently by numerous investigators[20,22-25]. Additional studies suggested that supplementation with the precursor of NAD, L-glutamine, may further enhance the NAD redox system[26].

With 30 grams of L-glutamine supplementation, there was improvement in NAD redox potential in 7 out of 7 patients. In addition, there were consistent reports of improved general clinical condition in such areas as energy level and chronic pain levels [10]. Furthermore, a short observation period of approximately 3 months suggested a decrease in the incidence of vaso-occlusive painful crises among those patients.

On the basis of these results, we proceeded to study the effect of L-glutamine therapy on endothelial adhesion of sickle RBC. As noted in the data presented here, in both cross-sectional and longitudinal studies, there is an indication that L-glutamine therapy improves the endothelial adhesion of sickle RBC.

In the cross-sectional study, patients who were already on the L-glutamine therapy were compared to those who have never been treated with L-glutamine. The adhesion in the untreated group was elevated as expected, which was consistent with previously published reports. On the other hand, the adhesion for the treatment group was very similar to those of normal control.

To confirm the cross-sectional data, the prospective study was conducted on 5 sickle cell anemia patients who were treated for 4 to 8 weeks. The results were consistent among each patient studied. With the therapy, each patient had significant improvement in endothelial adhesion while there was no improvement in the control patient who was not treated. The stimulants for endothelial cells were taken from the methods used in the previous studies by Kalra and collegues[13]. Their method is well established for static assay and based on their data, we felt their methods were applicable in our project. Adhesion assay for the cross-sectional study and longitudinal studies were conducted at two separate institutions but using same agents to stimulate the endothelial cells. The purpose of utilizing two expert sites for two parts of study was to improve objectivity by demonstrating that consistent data can be obtained even when assays were conducted by unbiased experts at multiple independent sites.

In the studies, we could not verify the increase in RBC survival duration as neither reticulocyte counts nor hemoglobin levels were not changed significantly. However, improvement in endothelial adhesion does not have to automatically result in increase in RBC survival. As these patients are asplenic, irreversibly sickled RBC are not rapidly removed and they will contribute to hemoglobin and hematocrit levels. Also, it is possible that with larger study with longer duration, there may be a change in these parameters with statistical significance. In general morphology of RBC appeared to have improved [see additional file 1 and 2].

Adhesion was assessed using a static endothelium-RBC adhesion system rather than a dynamic assay under conditions of flow over time. The decision to use the static assay was based on previous reports including that of the seminal article by Hebbel and colleagues[8,12,27]. In addition, as noted by Setty and others, the static assay may better simulate the interaction of sickle RBC and endothelium where microvessel flow may be slow and intermittent [27-29]. Obviously, there are also data suggesting that the dynamic endothelium-RBC adhesion system may better represent the *in vivo *activity. Such data favoring flow based assay may be found in the studies by Libowsky and colleagues, demonstrating with intravital microscopy that sickle RBC that became adherent during stasis created by a pressure cuff, dislodged when the cuff was removed and the flow resumed[30,31]. It is doubtful that there is an *in vitro *system that will perfectly represent the *in vivo *process. Rather, it is likely that both static and dynamic assays have roles in the investigation of sickle cell pathophysiology.

At present, the exact mechanism by which L-glutamine effectively decreases the endothelial adhesion of sickle RBC is not clear. However, there is evidence suggesting that one of the ways L-glutamine may benefit sickle RBC is by improvement of NAD redox potential[10]. This may prevent oxidant damage to RBC which may result in stimulation of inflammation and expression of adhesion molecules. In addition, L-glutamine may provide much needed energy and building material to maintain the integrity of sickle RBC. It has been reported that sickle cell children require increased amino acid consumption, especially glutamine[32].

## Conclusion

L-glutamine is an inexpensive compound widely consumed as part of a normal diet or as a dietary supplement. There is essentially no major adverse effect when used as an oral agent both in a research setting and non-research setting [33-36]. The data gathered so far including the ones presented in this article suggest that there is a physiological basis for the potential clinical benefit of L-glutamine in management of sickle cell disease patients.

## List of abbreviations

NAD = Nicotinamide Adenosine Dinucleotide

RBC = Red Blood Cells

HUVEC = Human Umbilical Vein Endothelial Cells

LPS = Lipopolysaccharide

BSA = Bovine Serum Albumin

HBSS = Hank's Buffered Saline Solution

HEPES = 4-(2-hydroxyethyl)-1 – piperazineethanesulfonic acid

EGM = Endothelial Cell Growth Medium

SEM = Standard Error of Mean

## Competing interests

YN and KRT have proprietary interest in the product tested in this study.

## Non-financial competing interests

None

## Authors' contributions

YN is the primary investigator and senior author. YN conceived of the study, designed and coordinated the study. YN also drafted the manuscript. NMM and YMS performed the endothelial adhesion assays and coordinated with SHE and VKK in the analysis and interpretation of data. SHC, DAA, MAS and JM were involved in acquisition of patient data as well ad the performance of various assays. CSJ and KRT have been involved in the conception of the study as well as in supervising composition of manuscript. All authors read and approved the manuscript.

## Pre-publication history

The pre-publication history for this paper can be accessed here:



## References

[B1] Hamdallah M, Bhatia AJ (1995). Prevalence of sickle-cell trait in USA adolescents of Central American origin. Lancet.

[B2] Weissman AM (1994). Preventive health care and screening of Latin American immigrants in the United States. J Am Board Fam Pract.

[B3] Diaz-Barrios V (1989). Newborn screening for sickle cell disease and other hemoglobinopathies. New York's experience. Pediatrics.

[B4] Frenette PS (2002). Sickle cell vaso-occlusion: multistep and multicellular paradigm. Curr Opin Hematol.

[B5] Perronne V, Roberts-Harewood M, Bachir D, Roudot-Thoraval F, Delord JM, Thuret I, Schaeffer A, Davies SC, Galacteros F, Godeau B (2002). Patterns of mortality in sickle cell disease in adults in France and England. Hematol J.

[B6] Wierenga KJ, Hambleton IR, Lewis NA (2001). Survival estimates for patients with homozygous sickle-cell disease in Jamaica: a clinic-based population study. Lancet.

[B7] Barabino GA, McIntire LV, Eskin SG, Sears DA, Udden M (1987). Endothelial cell interactions with sickle cell, sickle trait, mechanically injured, and normal erythrocytes under controlled flow. Blood.

[B8] Hebbel RP, Yamada O, Moldow CF, Jacob HS, White JG, Eaton JW (1980). Abnormal adherence of sickle erythrocytes to cultured vascular endothelium: possible mechanism for microvascular occlusion in sickle cell disease. J Clin Invest.

[B9] Vargas FF, Blackshear GL (1982). Vascular resistance and transit time of sickle red blood cells. Blood Cells.

[B10] Niihara Y, Zerez CR, Akiyama DS, Tanaka KR (1998). Oral L-glutamine therapy for sickle cell anemia: I. Subjective clinical improvement and favorable change in red cell NAD redox potential. Am J Hematol.

[B11] Zerez CR, Lachant NA, Lee SJ, Tanaka KR (1988). Decreased erythrocyte nicotinamide adenine dinucleotide redox potential and abnormal pyridine nucleotide content in sickle cell disease. Blood.

[B12] Kalra VK, Ying Y, Deemer K, Natarajan R, Nadler JL, Coates TD (1994). Mechanism of cigarette smoke condensate induced adhesion of human monocytes to cultured endothelial cells. J Cell Physiol.

[B13] Wali RK, Jaffe S, Kumar D, Kalra VK (1988). Alterations in organization of phospholipids in erythrocytes as factor in adherence to endothelial cells in diabetes mellitus. Diabetes.

[B14] Sugihara K, Sugihara T, Mohandas N, Hebbel RP (1992). Thrombospondin mediates adherence of CD36+ sickle reticulocytes to endothelial cells. Blood.

[B15] Hebbel RP, Schwartz RS, Mohandas N (1985). The adhesive sickle erythrocyte: cause and consequence of abnormal interactions with endothelium, monocytes/macrophages and model membranes. Clin Haematol.

[B16] Ohnishi ST, Horiuchi KY, Horiuchi K (1986). The mechanism of in vitro formation of irreversibly sickled cells and modes of action of its inhibitors. Biochim Biophys Acta.

[B17] Hebbel RP, Eaton JW, Steinberg MH, White JG (1982). Erythrocyte/endothelial interactions in the pathogenesis of sickle-cell disease: a "real logical" assessment. Blood Cells.

[B18] Sultana C, Shen Y, Rattan V, Johnson C, Kalra VK (1998). Interaction of sickle erythrocytes with endothelial cells in the presence of endothelial cell conditioned medium induces oxidant stress leading to transendothelial migration of monocytes. Blood.

[B19] Inwald DP, Kirkham FJ, Peters MJ, Lane R, Wade A, Evans JP, Klein NJ (2000). Platelet and leucocyte activation in childhood sickle cell disease: association with nocturnal hypoxaemia. Br J Haematol.

[B20] Rattan V, Sultana C, Shen Y, Kalra VK (1997). Oxidant stress-induced transendothelial migration of monocytes is linked to phosphorylation of PECAM-1. Am J Physiol.

[B21] Zerez CR, Lachant NA, Lent KM, Tanaka KR (1992). Decreased pyrimidine nucleoside monophosphate kinase activity in sickle erythrocytes. Blood.

[B22] Klings ES, Farber HW (2001). Role of free radicals in the pathogenesis of acute chest syndrome in sickle cell disease. Respir Res.

[B23] Anastasi J (1984). Hemoglobin S-mediated membrane oxidant injury: protection from malaria and pathology in sickle cell disease. Med Hypotheses.

[B24] Wetterstroem N, Brewer GJ, Warth JA, Mitchinson A, Near K (1984). Relationship of glutathione levels and Heinz body formation to irreversibly sickled cells in sickle cell anemia. J Lab Clin Med.

[B25] Beretta L, Gerli GC, Ferraresi R, Agostoni A, Gualandri V, Orsini GB (1983). Antioxidant system in sickle red cells. Acta Haematol.

[B26] Niihara Y, Zerez CR, Akiyama DS, Tanaka KR (1997). Increased red cell glutamine availability in sickle cell anemia: demonstration of increased active transport, affinity, and increased glutamate level in intact red cells. J Lab Clin Med.

[B27] Setty BN, Kulkarni S, Stuart MJ (2002). Role of erythrocyte phosphatidylserine in sickle red cell-endothelial adhesion. Blood.

[B28] Rodgers GP, Schechter AN, Noguchi CT, Klein HG, Nienhuis AW, Bonner RF (1984). Periodic microcirculatory flow in patients with sickle-cell disease. N Engl J Med.

[B29] Wun T, Paglieroni T, Field CL, Welborn J, Cheung A, Walker NJ, Tablin F (1999). Platelet-erythrocyte adhesion in sickle cell disease. J Investig Med.

[B30] Lipowsky HH, Sheikh NU, Katz DM (1987). Intravital microscopy of capillary hemodynamics in sickle cell disease. J Clin Invest.

[B31] Lipowsky HH, Williams ME (1997). Shear rate dependency of red cell sequestration in skin capillaries in sickle cell disease and its variation with vasoocclusive crisis. Microcirculation.

[B32] Salman EK, Haymond MW, Bayne E, Sager BK, Wiisanen A, Pitel P, Darmaun D (1996). Protein and energy metabolism in prepubertal children with sickle cell anemia. Pediatr Res.

[B33] Garlick PJ (2001). Assessment of the safety of glutamine and other amino acids. J Nutr.

[B34] Ziegler TR, Bazargan N, Leader LM, Martindale RG (2000). Glutamine and the gastrointestinal tract. Curr Opin Clin Nutr Metab Care.

[B35] Hasebe M, Suzuki H, Mori E, Furukawa J, Kobayashi K, Ueda Y (1999). Glutamate in enteral nutrition: can glutamate replace glutamine in supplementation to enteral nutrition in burned rats?. JPEN J Parenter Enteral Nutr.

[B36] Ziegler TR, Benfell K, Smith RJ, Young LS, Brown E, Ferrari-Baliviera E, Lowe DK, Wilmore DW (1990). Safety and metabolic effects of L-glutamine administration in humans. JPEN J Parenter Enteral Nutr.

